# Three week dietary intervention using apricots, pomegranate juice or/and fermented sour sobya and impact on biomarkers of antioxidative activity, oxidative stress and erythrocytic glutathione transferase activity among adults

**DOI:** 10.1186/s12937-016-0173-x

**Published:** 2016-05-12

**Authors:** Mostafa Gouda, Amr Moustafa, Laila Hussein, Mohamed Hamza

**Affiliations:** 1 Department of Nutrition & Food Sc, National Research Center, Giza, Dokki Egypt; 2 Department of Biochemistry, Faculty of Agriculture, Cairo University, Giza, Egypt; 3 Department of Agronomy, Faculty of Agric, Cairo University, Giza, Egypt

**Keywords:** Pomegranate juice, Sobya, Human trial, Antioxidative activity, Oxidative stress, Erythrocytic glutathione- S-transferase

## Abstract

**Background:**

The beneficial effects of the polyphenol (PP) rich fruits and Lactic acid bacteria fermented foods had been reported as cost-effective strategies for health promotion. Randomized controlled trial was designed to test the hypothesis that daily intake of polyphenol rich pomegranate juice (PGJ) or/ and lactic acid bacteria fermented sobya (FS) improved selected biomarkers of relevance to heath status.

**Methods:**

The design of the human trial consisted of 35 healthy adults, who were distributed to 5 equal groups; The first group served as control and received no supplements; the second group received fresh apricot fruits (200 g); the third (PGJ) (250 g), the fourth a mixture of PGJ (150 g) and FS (140 g) and the fifth group received (FS) (170 g). The supplements were served daily between 5 – 6 pm for 21 days. Blood and urine samples were collected at days zero and 22 of the dietary intervention. The supplements were analyzed chemically for (PP) contents and total antioxidative activities and microbiologically for selected bacteria and yeast counts. The blood samples were assayed for plasma antioxidative activities and for erythrocytic glutathione transferase activity (E-GST). Urine samples were analyzed for the excretions of total PP, antioxidative activity and thiobarbituric acid reactive substances (TBARS). Statistical analysis: Two way analysis of variance (ANOVA) was conducted and included the main effects of treatment, time and treatment x time interaction.

**Results:**

Daily intake of (PGJ) for 3 weeks significantly increased the plasma and urinary anti-oxidative activities and reduced the urinary excretion of (TBARS). Daily intake of (FS) for 3 weeks increased only (E-GST) activity. Daily intake of a mixture of PGJ and (FS) was also effective.

**Conclusions:**

The daily intakes of PGJ and/ or (FS) affected positively selected biomarkers of relevance to health status. These functional foods have potential implication for use as bio-therapeutic foods.

**Trial registration:**

The study was approved by the research ethical committee of the Ministry of Health & population, Egypt. The trial registration - the unique identifying number. (REC) decision No 12-2013-9, which complied with the Declaration of Helsinki guidelines (2004). The protocol was fully explained to all subjects and written informed consent was obtained before their participation in the trial.

## Introduction

Under normal physiological conditions, all cells in the body are exposed chronically to oxidants from both endogenous and exogenous sources; yet the intracellular “redox buffer” mechanism provides significant protection mainly by the antioxidant network [1]. Disturbance in the prooxidant- antioxidant balance in favor of the former leads to what is known as oxidative stress [2]. This oxidative stress and reactive oxygen species (ROS) can cause damage to DNA, proteins and lipids an d end up with an epidemic of non communicable chronic human diseases [3–5]. The prevalence of NCD are at escalating in Egypt due to activation of 64 genes involved in inflammation [6, 7] and other modifiable risk factors [8]. Medical and pharmacologic chemotherapeutic agents were reported to reduce cardio vascular mortality among individuals at risk, but they may induce oxidative stress, which increases to an invasive stage with disease progression [9]. Plant polyphenols possess the ideal chemical structure for free radical scavenging activity and their in vitro antioxidative activities are more effective than tocopherols and ascorbate [10]. Designing effective preventive strategies using naturally occurring phytonutrients aiming at reducing oxidative stress is one of the cost-effective strategies to move people’s lifestyle toward healthier behaviors [11].

Pomegranate is a popular fruit grown in Egypt with annual production of approximately 130000 tons [12]. Natural unprocessed pomegranate juice (PGJ) is superior to commercial juices in their polyphenol (PP) contents with mean levels of 421 and 382 mg per 100 ml, respectively. Another investigation reported respective (PP) concentrations of 139 mg gallic acid equivalent (GAE) per 100 ml juice and may reach over 200 mg GAE/ 100 ml, when the other phenolic compounds; anthocyamin, ellagitannins, and tannin punicalagin were included [13]. Dietary pomegranate intake in human trials elevated urinary excretion of the above mentioned phenolic metabolites, which are the bioactive constituents responsible for more than >50 % of the antioxidative capacity activity of the juice [14, 15] and are the biomarkers linked to health promotion [16]. Measurement of GST activity had been recommended for the evaluation of protective treatment in trials considering antioxidant strategies. The expression of the phase II hepatic glutathione *S*-transferase was activated in the liver cells of animals following feeding pomegranate anthocyanins flavonoids. The molecular mechanism was related to activation of antioxidant response element (ARE) upstream of genes that regulate the expression of GST [17].

Specific strains of lactic acid bacteria (LAB) possess also antioxidative ability, scavenge reactive oxygen species and chelate metal ions, which provide protection against oxidative stress and lipid peroxidation [18, 19]. The regular intake of LAB fermented foods such as sourdough [20], water kefir [21, 22] and sour sobya [23] containing specific strains of (LAB) with antioxidative capacity activity, contribute to their health effects. Furthermore, the oral administration of some strains of LAB (*L plantarum*) to humans was capable to break down phenolic acids and hydrolysable tannins into phenolic metabolites that are more easily absorbed in the body and enhancing the gut antioxidative effects [24].

In vivo and ex vivo functional biomarkers such as thiobarbituric acid reactive substances (TBARS) are developed for assessing exposure to antioxidant and to lipid oxidation and oxidative stress status [25].

The objective of the present study is to assess the effectiveness of dietary intervention with apricots (AP), pomegranate juice (PGJ), LAB fermented sour sobya (FS) or mixtures of PGJ and FS by healthy adults on biomarkers of antioxidative capacity activity (AEAC), oxidative stress (TBARS) and phase II glutathione-S-transferase enzyme activity [E- GST].

## Subjects and methods

A Randomized controlled trial (RCT) was conducted on 35 healthy Egyptian adults (25 men and 10 women) between the ages of 21– 35 years (mean age 27.8 ± 0.96 years). The study received the approval from the research ethical committee (REC) decision No 12-2013-9, Ministry of Health & population, Egypt, which complied with the Declaration of Helsinki guidelines (2004). The protocol was fully explained to all subjects and written informed consent was obtained before their participation in the trial.

Inclusion criteria were healthy adults aging 21 – 35 years. Exclusion criteria at the time of the screening were: History of diabetes, hypertension, heart disease, endocrine disorders, abnormal blood chemistry profile, fasting LDL-cholesterol concentration > 3.37 mmol/L, fasting triacylglycerol concentration > 3.39 mmol/L, smoking, use of antioxidant, vitamin supplements or having taken part in any dietary intervention trials. Female subjects were neither pregnant nor lactating. The volunteers were instructed to continue to eat their normal diet and not to alter their usual dietary or fluid intake with the exception of the foods and beverages that are high in dietary flavonoids, such as purple grapes, cocoa and chocolate during the entire three week dietary intervention trial. Also As a Muslim country red wine drinking is unknown to almost all of the youths, because its drinking is prohibited by religion. Also Egypt is not a coffee drinking country. Volunteers who met the initial inclusion/exclusion criteria took part in the study and there were no withdrawals.

Participant characteristics are summarized in Table 1. Supplements Apricots (AP) (family *Rosaceae; Prunus Armenia*) and pomegranate (family *Punicaceae; Punica granatum L.*) were purchased in batches from El Obour market. AP were stored immediately in packages each containing 200 g net (AP) and were saved in the refrigerator at 4 °C. Pomegranates were picked by hand, washed and stored in tanks. The fruit was crushed and squeezed in an electric juicer (Brand, Germany) and the juice (PGJ) (approximately 30 % of the fruit weight) was packed immediately in 250 ml or 100 ml portions in air tight polyethylene bottle with screw caps and frozen at -200 °C. Lactic acid bacteria fermented sour sobya (FS) was purchased from the retail market (El Rahmany brand) once weekly and saved in the refrigerator.

**Table 1:** Baseline characteristics of the study subjects

Parameter	Unit	x̄ ±SE	Median	Q1	Q3
Age	years	27.8±0.96	27.00	24.00	29.75
BMI	Kg.m^-1^	23.7±0.58	23.57	21.48	25.98
Estimated energy intake	Kcal/d	1969±138.78	1903.0	1555	2262
Plasma Antioxidant activity	AEAC/100ml	3.79±0.33	3.37	2.92	4.27
Erythrocyte GST Activity	IU/g Hb	4.65±0.46	4.09	2.73	6.23
Urinary polyphenols	GAE/mg creat	11.45±2.57	6.96	4.89	12.59
Urinary Antioxidant activity	AEAC/mg creat*	7.21±0.84	6.46	4.04	8.55
Urinary TBA	μg/mg creat	114.75±17.37	83.30	47.76	141.71

x̄ ±SE: Mean ± standard Error, * : mmol ascorbic acid equivalent antioxidant capacity / mg creatinine),

GAE : gallic acid equivalent

### Design of the RCT

The volunteers were divided into five groups of equal size and each group consisted of 5 men and 2 women. The first group served as control and received no supplements; the second group received fresh (AP) fruits (200 g); the third (PGJ) (250 g), the fourth a mixture of PGJ (150 g) and FS (140 g) and the fifth group received (FS) (170 g). The portions were reasonable dose to eat or to drink over the course of a day and the volunteers were given instructions to consume the portion between 5 – 7 pm.

### Dietary assessment

Three-day food diaries (including two weekdays and one weekend day) were completed by the participants to monitor the subjects’ dietary compliances and assess their nutrient intakes. Dietary intake were calculated using a computer-aided nutritional analysis program (Nutri survey, Seoul, Korea).

### Venous blood collection

One day before starting the trial and on day 22, Blood was taken into vacutainer tubes containing citrate, under quality control. The plasma was separated from blood by centrifugation in a cooling centrifuged at 3500 rpm for 10 min. Collected plasma were stored at -200 °C until analysis. The red blood cells were washed with cold physiological saline and the packed red blood cells were stored at – 200C.

Urine samples were collected from all volunteers at day zero and day 22 and saved frozen at -20 °C.

### Blood and urine collection and storage

Overnight fasted blood samples (3 ml) were collected one day before starting the dietary intervention and after 3 weeks of the dietary intervention (Day 22). All blood samples were collected from the left antecubital vein between 8:00 and 9:00 am in tubes containing sodium citrate. The plasma was separated from blood cells by centrifugation at 3000 rpm for 15 min at 4o C and the separated plasma was stored at -700 C for later biochemical analysis. The red blood cells (RBCs) were washed using cold physiological saline solution and stored at -200C. Urine samples were collected in the early morning after the subjects had fasted 10–12 h both on −1, and +21 d dietary intervention and aliquots (2 mL) were immediately frozen at −20 °C for biochemical analysis.

### Laboratory investigations

#### Determination of total polyphenols

The solid-phase extraction (SPE) Cartridge (60 mg) Strata-C18 (Phenomenex,Torrance,CA, USA); Folin Ciocalteu reagent (Catalog No47641; Sigma, USA). MilliQ ultrapure water (Millipore Iberica, Madrid, Spain). All of the analytical steps were carried out in dim light. The PPs of AP and PGJ were extracted vigorously from 4 g AP or 10 g PGJ with 5 ml distilled water then with 20 ml absolute MetOH. After centrifugation in the cold, the supernatant was aspirated and concentrated in vaccuo to 4 ml [26]. Clean up step on Waters 3 ml HLB (60 mg) cartridges were equilibrated with the successive addition of absolute methanol (1 ml) and H_2_O (1 ml). Aliquots of the methanolic fruit extracts (2 ml) were loaded on the activated SPE; washing with the successive addition of H_2_O (10 ml) and 5 % aqueous MetOH (1 ml). The PP containing fraction was eluted after the addition of 5 ml absolute MetOH. The elute was concentrated under a stream of nitrogen to 2 ml. Aliquots (20 μl (were taken for the analysis of PP using the Folin Ciocalteau colorimetric method [27] and the results were expressed as gallic acid equivalent (GAE) per g apricot or g PGJ.

### Measurement of total polyphenolic compounds in urine

Total polyphenols in the urine samples were assayed after the clean up step by the solid-phase extraction (SPE) as described earlier [28, 29]. Aliquots of the centrifuged urine samples (1 ml) were acidified with normal hydrochloric acid (17 μl) and were loaded onto an activated Waters Cartridge, preconditioned with 1 ml methanol and equilibrated with 1 ml of 1.5 mol/l aqueous formic acid. The SPE cartridge was washed twice with 2 ml of the same formic acid and 2 ml of water-methanol (95:5 by volume) to elute the impurities. Elution of the (PP) was completed with a mixture containing 1 ml of methanol containing 1 ml/l of formic acid (2 %)- methanol- H_2_O (2/60/38 v/v/v). Aliquots of the elute (500 μl) were pipetted into Eppendorf vials for the colorimetric determination of the (PP). Successive addition of Folin_Ciocalteu reagent (50 μl), followed by 20 % sodium carbonate (600 μl). The tubes were left in the dark for 60 min and then the absorbance of the developed color was measured at 765 nm against a blank. A blank and standard working solutions of gallic acid (0.215 mg/ml) were run in parallel. Urinary (PP) excretion was expressed as mg GAE per g of creatinine.

### Plasma antioxidative activity ascorbic acid equivalents (AEAC)

The assay is based on the measurement of the scavenging ability of antioxidants towards the stable DPPH (1,1-diphenyl-2-picrylhydrazyl) radical compound [30]. Aliquots of plasma samples (100 μl) were mixed with 400 μl methanol and after vortexing the tubes were centrifuged at 10,000 x g for 10 min at 4 °C. Aliquots of the clear protein free supernatant (20 μl) was added to 350 μl DPPH reagent (0.127 mol/L methanol) under vigorous shaking for 30 sec. The tubes were then incubated in the dark at room temperature for 30 min. The control was prepared as above without any extract, and MeOH was used for the baseline correction. Standard solutions of ascorbic acid at increasing concentrations (1- 5 μmol/ l ) were freshly prepared and treated as described above. Reduction of the absorbance at 515 nm was measured using a UV-Vis spectrophotometer (Shimadzu-Germany). A standard curve was plotted for the relation between ascorbic acid concentrations and the absorbance at 515 nm. All experiments were carried out in triplicate. The radical scavenging activity (RSA) was expressed as the inhibition percentage using the following formula:

$$ \%\ \mathrm{R}\mathrm{S}\mathrm{A} = \left(\left(\mathrm{Abs}\ \mathrm{control}\ \hbox{-} \mathrm{Abs}\ \mathrm{sample}\right) \times 100\right)/\ \mathrm{A}\mathrm{bs}\ \mathrm{control} $$

The plasma antioxidative capacity activity was expressed as mmol antioxidative activity ascorbic acid equivalents (AEAC) per 100 ml plasma.

### Urinary antioxidative activity ascorbic acid equivalents (AEAC)

This was assessed using the DPPH equivalent, according to an adapted colorimetric procedure [31]. Aliquots of filtered urine samples (100 μl) were mixed thoroughly using a vortex mixer with 3 mL DPPH solution in methanol (0.05 g/L) and the tubes were kept in a water bath at 25 °C in dim light for 30 min for the complete development of the reaction. The absorbance of the yellow color was measured spectrophotometrically against a blank at 517 nm.

Three measurements were conducted for each extract. The control solution contained 100 μl of DI water and 3 mL of DPPH solution in methanol. Standard ascorbic acid of increasing concentrations (2-10 μM/L) was treated in a similar manner and run in parallel. The inhibition of free radical in percent (I %) was calculated according to the formula: [( A0 - A1) / A 0] x 100, whereby A0 is the absorbance of the control reaction (containing all reagents except the urine), and A1 is the absorbance of the urine sample. All measurements were carried out in triplicates. Urinary antioxidant activity was expressed as as mmol antioxidative activity ascorbic acid equivalents (AEAC) per mmol creatinine).

### Urinary thiobarbituric acid-reacting species (TBARS)

Lipid peroxidation (LP) was measured in the urine samples as malondialdehyde (MDA), which was assayed by the thiobarbituric acid reactive species (TBARS) as described earlier [32]. Briefly, aliquots (200 μl) of urine samples were mixed with 10 μl of 5 % butylated hydroxyl toluene in glacial acetic acid, followed by the addition of 300 μl of 0.5 % aqueous TBA solution. After vortexing, the tubes were incubated at 100ο C for 30 min and the absorbance of the developed pink color was recorded spectrophotometrically at 532 nm. Samples were blanked against reference cuvettes containing reagent without urine. The concentration of TBARS was calculated from the absorbance at 532 nm using the molar extinction coefficient of 156000. To control for urine concentration the data were normalized to urine creatinine concentration.

### Determination of urinary creatinine

Aliquots of the urine samples (3 μl) were pipetted into a 96-well plate for the determination of creatinine by the alkaline picrat method [33]. Aliquots (60 μl) of aqueous picric acid solution (10 g/l) and a 60 μl aliquot of sodium hydroxide (100 g/l) were added in sequence. After 15 min the intensity of the color was read at 500 nm in the ASYS Expert Plus multi-channel microplate reader (Biochrom Lt, Cambridge, UK). The concentration was calculated using a standard creatinine solution.

### Assay of erythrocytic glutathione-S-transferase activity (E-GST).

The principle of the assay is based on the conjugation of 1-chloro-2,4dinitro benzene (CDNB) with glutathione (GSH)

$$ \mathrm{CDNB} + \mathrm{G}\mathrm{S}\mathrm{H}\ \to\ \mathrm{CDNB}\ \hbox{--}\ \mathrm{S}\ \hbox{--}\ \mathrm{glutathione} $$

Aliquots (20 μl) of the washed erythrocytes were lyzed by mixing with 180 μl stabilizing reagent [34] and the tubes were kept in a thermo flask at -18 °C for 10 minutes. After thawing, 50 μL of the hemolysate was added to enzyme assay mixture containing 0.68 ml of distilled water and 200 μL phosphate buffer (100 mM phosphate buffer, K_2_HPO_4_/KH_2_PO_4_; pH = 6.5) and 20 μL CDNB (0.5 mM) pre- incubated at 25oC for 10 minutes. The enzymatic GST assay started immediately at 25 °C by adding 50 μL GSH (1.0 mM). And monitoring the rate of increase in absorbance at 340 nm for 10 minutes at 25 °C against a blank solution containing the reaction mixture, in which; the haemolysate was substituted with distilled water [35]. The specific E-GST activity is μmol glutathione transferred /min at 37 °C/g Hb, which is synonymous to IU/g Hb.

Hemoglobin concentration was determined in the hemolysate by the cyan- methaemoglobin reaction using Drabkin reagent [34] and the results were expressed in g/dL.

### Statistical analysis

Values are presented as means and SEM and differences with values <0.05 are considered significant. Two way ANOVA were conducted using the general linear model procedure to assess the differences in the effect of the four dietary supplements (Apricots, pomegranate, mixture of pomegranate – sour sobya or sour sobya) and control on outcome measures. The models included the main effects of treatment, two time points and treatment x time interaction. Fisher's least significant differences were used to account for multiple testing [36]. Pearson correlation was used in the correlation evaluations. All evaluations were performed with excel software (release 16) and MSTAT-C computer package [37].

## Results

Table 1 presents the baseline characteristics of the volunteers. At base line all participants excreted urinary total polyphenols, but the inter individual variation was quite high (4.89-12.59 mg GAE / 100 ml urine).

Composition of the four supplements served to the volunteers are presented in Table 2. Dietary supplements 1,2,3,4,5 provided daily 0, 1.52, 24.71, 9.89 and 0 mg total polyphenol per portion. Respective antioxidative activity ascorbic acid equivalents (AEAC) were 0, 5.70 ± 0.74, 11.35 ± 2.2, 11.37 ± 2.05 and 7.74 ± 1.33 as showed in Table 2. The 170 g portion of FS provided 5.1 × 10^9^ cfu total *Lactobaccilli* and 2.77 × 10^10^ cfu yeasts, while the respective counts were 4.5 × 10^9^ cfu total *Lactobaccilli* and 2.44 × 10^10^ cfu yeasts in the daily portion of the mixture of PGJ & FS supplement.

**Table 2:** Composition of the supplements

Parameter	Unit	Dietary supplements				
		Control	AP^*^	PGJ^*^	PGJ^*^ + FS^*^	FS^*^
		Portion served	Portion served	Portion served	Portion served	Portion served
Portion Size	g	--	200	250	100 +150	170
Total solids	g	--	27.47	17.75	48	40.01
Carbohydrate	g	--	22	32.75	59	51.1
Dietary Fiber	g	--	4	0.25	48.6	54
Energy	Kcal	--	100	135	290	263
*Lactobacillus, diverse*	cfu / serving size	--	--	--	4.5 × 10^9^	5.1 × 10^9^
*Yeast*	cfu /serving size	--	--	--	2.44×10^10^	2.77×10^10^
Total Polyphenols	mg* / portion g	--	32±0.24	519.1±8.75	207.65±3.5	--
Antioxidant activity	(AEAC)**	--	5.70±0.74	11.35±2.2	11.37±2.05	7.74±1.33

*: mg gallic acid equivalent (GAE) , Ap: Apricot, PGJ: Pomegranate juice, FS: Fermented sobya;

**: mmol ascorbic acid equivalent antioxidant capacity

The urinary total polyphenol excretion increased by approximately 9- fold excess from baseline level of 5.7 to 55.2 μg/mg creatinine following 3 week dietary intervention with PGJ (P < 0.01). The 3 week dietary intervention with the mixture of PGJ - FS increased urinary polyphenol excretion compared to the respective baseline level, but the difference didn't attain significant level (P > 0.05). Neither the control group nor the three week dietary intervention with AP change the urinary total polyphenol excretion compared to the respective baseline levels (P > 0.05) (Table 3).

**Table 3:** Initial and final mean urinary polyphenols, plasma and urinary antioxidative activity, urinary TBARS and erythrocytic GST activity among subjects receiving different dietary supplements

Parameter	Unit	Control			Apricot			Pomegranate			Pomegranat+Sobya			Sobya		
		Baseline	Final		Baseline	Final		Baseline	Final		Baseline	Final		Baseline	Final	
		x̄±SE	x̄±SE	P	x̄±SE	x̄±SE	P	x̄±SE	x̄±SE	P	x̄±SE	x̄±SE	P	x̄±SE	x̄±SE	P
Urinary polyphenol	GAE/mg creat	10.36±1.8	8.11±2.2		18.47±9.7	10.36±2.2		5.70±1.4	55.23±21.7	<0.05	10.40±3.2	21.62±7.3		11.84±6.2	9.86±1.8	
Urinary Antioxidant activity	AEAC/mg creat*	9.74±2.0	8.13±2.7		4.25±0.9	7.56±2.9		7.18±0.9	46.57±18.0	<0.05	10.90±2.4	20.25±3.9		3.89±0.9	10.30±2.3	
Urinary TBARS	μg/mg creat	83.04±12.1	75.17±15.3		68.82±30.9	50.19±7.7		173.93±44.8	51.48±8.2	<0.05	157.70±47.8	40.62±8.3	<0.05	82.77±27.8	29.97±4.4	
Plasma antioxidant	AEAC/100 ml	6.36±2.81	5.99±2.66		4.16±0.51	3.90±0.86		3.64±0.30	5.92±0.68	<0.05	2.78±0.11	4.49±0.58	<0.05	3.70±0.33	4.55±0.27	
E-GST Activity	IU/g Hb	5.94±3.3	5.45±4.1		4.64±1.2	3.97±0.8		4.73±1.0	8.34±1.0		4.56±1.0	6.90±1.0		4.26±0.5	7.21±0.8	<0.05

x̄±SE: Mean ± standard Error, * : mmol ascorbic acid equivalent antioxidant capacity / mg creatinine, GAE : gallic acid equivalent; Mean values are significantly different if the P-Values are less than 0.05 (P<0.05)

The three week dietary intervention with PGJ was associated with significant increase in the mean plasma antioxidative activity ascorbic acid equivalents (AEAC) compared to the respective baseline level (P < 0.05) and similar significant increase were obtained in the urinary AEAC (P < 0.05 ) compared with the respective baseline concentrations (Table 3). The three week dietary intervention with a mixture of PGJ and FS was associated with an increase in the plasma (AEAC) compared with the respective baseline level (P < 0.05). The 3 week dietary intervention with fermented sobya (FS), increased the plasma AEAC by 23 %, which did not attain significant level (P > 0.05), while the urinary increase in AEAC (131 %) compared to the respective baseline levels, but the difference didn't attain significant difference (0.1 > P > 0.05) (Table 3). Neither the control group nor the dietary intervention with AP changed the AEAC to any extent and the results were similar to those obtained with the respective base line levels (Table 3).

Urinary excretion of TBARS decreased by 63.5, 41.6 and 48.1 % at the end of the three week dietary intervention with PGJ (P < 0.05), mixture of PGJ & FS (P < 0.05) and FS (0.1 > P > 0.05), respectively compared to the respective baseline levels. Neither the control group nor the three week dietary intervention with apricots reduced the urinary TBARS concentration (P > 0.05) (Table 3).

The baseline Erythrocytic glutathione-S- transferase activity (E-GST) activity averaged 4.65 ± 0.46 IU/g Hb. The mean E-GST increased significantly after the 3 week dietary intervention with fermented sobya (FS) compared with the respective base line activity (P < 0.05). The increases in the enzymatic activities among the four other groups didn't attain significant level (P > 0.05).

Table 4 presents the two way ANOVA showing no significant differences between individuals. The ANOVA test reveals high stastistical significant differences in the other sources of variations, *i.e* dietary supplements, time points and interaction between dietary supplements and time points. Fisher's least significant differences were used to account for multiple testing

**Table 4:** Mean squares analysis of variance for E-GST activity, plasma anti-oxidative activity, urinary anti-oxidative activity, urinary TBA and urinary polyphenol excretions under five dietary supplements

Source of variation	df	E-GST	Plasma Antioxidant	Urinary Antioxidant	Urinary TBA	Urinary polyphenol
Replicates	**6**	19.00	0.36	123.16	7508.8	642.51
Dietary Supplement (DS)	**4**	9.04**	13.96**	1052.62**	8423.2*	993.17
Time point (T)	**1**	41.84**	11.87**	2261.58**	71153.3**	1640.05*
Interaction (T × DS)	**4**	13.92**	4.64**	916.08**	10122.7*	1911.84**

* and ** denote significance at 5% and 1% probability level, respectively

Very high positive significant correlation coefficients were obtained between urinary total polyphenols concentration and urinary AEAC (r =0.96,P < 0.000). Inverse significant correlations were also found between the excretions of urinary total polyphenols total polyphenols concentration and urinary TBARS excretion (Table 5).

**Table 5:** Correlation coefficients between Percentage change(%) of biochemical plasma antioxidative, urinary polyphenol, urinary antioxidant and urinary thiobarbituric acid

Biochemical parameter	Unit	Urinary polyphenol	Urinary antioxidant activity	Urinary TBARS
Urinary polyphenol	GAE/mg creat	1	0.93^***^	-0.31^*^
Urinary antioxidant activity	AEAC/mg creat*	0.93^***^	1	-0.28^*^
Urinary TBARS	μg/mg creat	-0.31^*^	-0.28^*^	1

*:P<0.05;**:P<0.01;***:P<0.001; (AEAC) *: mmol ascorbic acid equivalent antioxidant capacity/creatinine

## Discussion

The promotion of a healthy life style with non-pharmacological means is a preventive measure strategy and our work aimed at throwing light on the importance of carrying out dietary intervention trials in human beings with the PGJ and probiotic LAB fermented food products. The relationship between the polyphenol rich fruits and LAB fermented supplements and prevention of diseases in man is the topic of numerous investigations during recent years [38].

Antioxidant potency of commonly consumed PGJ was evaluated through several in vitro methodologies. Hundreds of Norwegian dietary plants were screened for antioxidative activity by automated FRAP assay, and pomegranate was the fruit with the highest antioxidative activity ascorbic acid equivalents (AEAC) with mean level of 11.3 mmol /100 g, while the respective value for apricot was 0.52 mmol /100 g [39]. Slightly higher antioxidative activities were reported for fresh PGJ with mean level of 15 AEAC / L, compared to the respective value of 12 – 13 AEAC for frozen PGJ [40].

The antioxidant capacity of PGJs obtained from eight Iranian cultivars were determined by the scavenging activity against (DPPH) showed that the cultivar Izmir 8 was with the highest scores with efficient concentration (EC 50) of 29.8 ± 2.9 ml PJ/ g of DPPH [41]. The above mentioned reported levels are higher than the respective mean level of 4.5 AEAC per Liter PGJ obtained in the present study.

The bioavailability of pomegranate flavonoid is influenced by “LADME” [42]. The concept LADME stands for liberation from the food matrix (bioaccessibility), absorption, distribution, metabolism and excretion [43]. The dietary polyphenols escape absorption in the small intestine and are degraded in the colon under the catalytic action of the enormous number of colonic microbiota amount to 1014 colony forming units.

The first step in polyphenol degradation involves the release of aglycones by microbial glycosidases, which enhances their absorption. Plasma ellagic acid (EA) peaked after one hour of oral ingestion of 180 mL PGJ with mean concentration of 31.9 ng / mL plasma [14]. Identical results were obtained following the daily consumption of capsules (800 mg) containing 330.4 mg pomegranate punicalagins and 21.6 mg EA [44]. According to the authors, the (EA) peak reached 33.8 ± 12.7 ng/mL plasma. This finding presents evidence that pome granate ellagic acid (EA) was absorbed in humans. The increase in pomegranate ellagic acid (EA) was associated with significant increases in plasma AEAC averaging 31.8, 1.62 and 1.43 %, after 0.5, one and two hours post ingestion, respectively [44]. Furthermore, urinary urolithin metabolites persisted for 48 hours after PGJ ingestion [45].

In the present study, the three week dietary intervention with PGJ was quite appropriate to raise and maintain the body pools of phenols. The high AEAC of plasma and urine of our subjects is in good agreement with other studies reporting high plasma antioxidant capacity in human volunteers after PGJ consumption [44].

It is suggested that the gastrointestinal tract plays an important role in the first-pass metabolism of dietary flavonoids [46], whereby, during phase I metabolism, intestinal bacteria reductases convert pomegranate flavonoids into the urolithins and enabling reuptake [47]. Urolithin A and urolithin B are the PGJ metabolites detected in the plasma of volunteers following 5 day intakes of one liter of PGJ daily containing 4.37 g/L punicalagins and 0.49 g/L anthocyanins [48]. Their persistence in plasma and tissues suggest that they account for some of the health benefits of PGJ [14]. Specific strains of L. plantarum possess enzyme tannase (tannin acyl -hydrolase) and are able to degrade hydrolyzable tannins and tannic acid, to the monomeric antioxidant derivatives gallic acid and pyrogallol [49]. Eubacterium ramulus is one of the key colonic bacteria implicated in the biotransformation of the polyphewnolic compounds quercetin, apigenin and naringenin [50]. In phase II metabolism, polyphenols and their reductive metabolites are then conjugated with glucuronic acid and sulfate. by intestinal microsomes [47].

Erythrocytic glutathione-S-transferase (E-GST) designated GST3 or GSTı belongs to phase II metabolizing enzyme superfamily involved in the cellular detoxification of both xenobiotic and endobiotic compounds [51]. This enzyme serves to scavenge free radicals that are produced from oxidative stress by actively transporting GSH- xenobiotic conjugates. Induction of E-GST by expression had been implicated in protection of blood stream and other target cells from genotoxic insults [52]. One study reported no changes in the activities of E-GST following treatment with pomegranate polyphenols, although the concentrations of the lipid peroxidation marker malondialdehyde was reduced [53]. Our results are in agreement with this report, since the increase in E-GSH activity was not significant following the 3 week dietary intervention with PGJ, yet the reduction in lipid peroxidation marker malondialdehde was tremendous. Significant increase in the E –GST activity was found only after the three week dietary intervention with fermented sobya (t = 2.27, P < 0.05). This finding suggests that the sobya micro-organisms (LAB and/ or yeast) potentially modulated the expression of GST genes related to biotransformation. This finding supports the important role of fermented products in primary chemo- prevention. Reinforcing the activity of GST protects target cells [54]. Similar finding was reported with the complex fermentation of inulin-type fructans which favourably modulate the expression of genes related to biotransformation in carcinoma cells [55].

Urinary excretion of either free aglycones or conjugated polyphenols in humans are the valid biological markers of exposure and are more affordable approach in epidemiological studies and in metabolic studies [56, 57]. Repeated consumption of PP rich food sources resulted in the urinary excretion of 111 metabolites and about 160 aglycone metabolites originating from various classes of dietary polyphenols and maximum excretion rates occurred 3-4 days after juice ingestion [58, 59]. More than 80 urinary polyphenol metabolites could be identified nowadays with high predicting ability of food intake (food metabolome) within the population of 4 European countries [57]. The development of novel analytic methods is now essential to measure large sets of polyphenols biomarkers and evaluate the role of these metabolites in disease prevention. The biomarkers are indicator of responses to dietary intervention that can aid in understanding the prediction of treatment [60]. In the present study, daily intake of 250 ml PGJ totaling 5.25 liters per 21 days increased the mean urinary polyphenol excretion (P < 0.05) from 6 GAE at day 0 to 55 GAE/mg creatinine at day 22. All other supplements failed to increase urinary PP excretion to a significant level (P > 0.05). In both laboratory and clinical studies, the pomegranate combats numerous pathological changes associated with cardiovascular disease [61, 62]. Pomegranate fights cardiovascular disease by reducing oxidative stress, inhibiting the oxidation of potentially harmful LDL [63], quenching free radicals and supporting the synthesis and activity of nitric oxide [64, 65]. Oxidation protection actions of dietary polyphenols include reactive species scavenging, enzyme modulation to interference with cell signaling and oxidative stability [66]. The chemo preventive action of PGJ was attributed to the modulation of cellular signaling processes including nuclear factor-kappa-B (NF-кB) and activator protein-1(AP-1) DNA binding [67]. Separation of individual polyphenols from the crude PGJ were interpreted on the basis that pretreatment of PGJ inhibited NFκB activation, AKT activity and expression of COX-2 in HT-29 cells [67].

It is not clear, if the antioxidants in dietary plants polyphenol can explain all of the protective effect against oxidative stress-related chronic diseases. Synergistic effect between the supplements PGJ & LAB - fermented sobya biopeptides may be acting in coordination to protect against oxidative stress events. In agreement with our finding, apricot cultivars grown in Spain showed low contents of bioactive compounds with mean polyphenol concentration of 179.8 mg GAE/100 g [68]. Interestingly, heat-processed apricots have excellent antioxidative properties due to the high melanoidins content. Derived melanoidin is responsible for the significant elevation of antioxidant capacity and protecting endothelial cells from hydrogen peroxide - induced oxidative mitochondrial damage and cell death [69]. Evidence obtained from the present trial is based on a healthy adult population group, may not be generalizable to individuals with a disease or specific risk factors. Inability to apply molecular markers or food-specific signatures such as metabolomic analysis is a further limitation due to protocol limitation.

## Conclusion

The nutrition intervention with mixtures of PGJ and probiotic fermented sobya had synergistic positive effects. PGJ enhances the antioxidant status in humans by decreasing the oxidative damage in lipids by decreasing urinary TBARS and improving the antioxidant mechanisms; while probiotic fermented sobya stimulates erythrocytes GST activity. The present study demonstrated that designing effective preventive strategies aiming at reducing the risk of chronic diseases is a cost effective safe initiative in contrast to the action of drugs developed by the pharmaceutical industry. Quantification among individuals of biologically effective pomegranate dose, interacting with critical cellular targets for curing or preventing various illnesses warrants further investigations. Research concerning increased knowledge on the depth of the role of nutrients in the health promotion and.
